# Avoiding Real Harm but False Good: The Influence Mechanism of Political Relations on the Effectiveness of Environmental Regulation Policies

**DOI:** 10.3390/ijerph192315953

**Published:** 2022-11-30

**Authors:** Bin He, Mengzhen Qi, Ning Wang, Zhenhua Zhang

**Affiliations:** 1 School of Public Policy and Management, Tsinghua University, Beijing 100084, China; 2 School of Political Science and Public Administration, Huaqiao University, Quanzhou 362021, China; 3 School of Social and Public Administration, East China University of Science and Technology, Shanghai 200237, China; 4 Institute of Green Finance, Lanzhou University, Lanzhou 730000, China

**Keywords:** environmental regulation, political connections, policy effect, regulatory severity, regulatory certainty

## Abstract

In environmental authoritarian countries, environmental pollution control relies on government environmental regulation. Theoretically, the certainty and severity of environmental regulation are the key factors in achieving its political goals. According to regulatory space theory, an effective regulatory system needs regulatory power and resources. However, the effectiveness of regulation may be decreased by the desperate need for resources, and the regulated enterprises can also affect environmental regulation through their information advantage and social networks. This paper focuses on how local environmental regulation can be achieved under these conditions. The analysis is conducted from two perspectives: the deterrence effect of punishment and the political connections maintained by enterprises. An empirical test was conducted by analyzing the research data from the 12th China Private Enterprise Survey in 2016. The study found that the severity of punishment is a mediator of environmental regulation in the promotion of enterprises’ investment in pollution control, and that it is moderated by the political relationships of enterprise managers. Compared with counterparts that have a less substantial political network, enterprises with more political networks may feel more pressure from environmental regulation policies, which leads to higher pollution fines. However, the promotion effect of environmental fines on an enterprise’s investment in pollution control is weakened due to its political relationships, thus weakening the effectiveness of the punishment. This paper clarifies the mechanism through which political connections weaken the effect of environmental regulation: political connections encourage firms to avoid real harm and do false good. Regulation is supposed to use fines as an incentive to improve the environment, but politically connected companies are more willing to pay fines (doing false good), than to invest in cleaning up pollution (avoiding real harm).

## 1. Introduction

Government regulation is key to combating environmental pollution in most industrial countries, and strict environmental regulation is important for improving environmental quality (to avoid repetition of what has been observed in many countries). Many studies have shown that the government’s strict environmental monitoring and law enforcement are the first driving force encouraging enterprises to improve environmental pollution behavior [1,2]. In terms of effect, governmental environmental regulation has a significantly higher restraining effect on the environmental behavior of enterprises than non-governmental regulatory factors, such as associations, communities, and the media [3,4]. In the actual survey, many business managers even use government environmental regulations as the only source of motivation to cut down on corporate pollution behavior. Therefore, how can effective environmental regulation be achieved in the case of multiple systems of government? Existing research argues that the institutional arrangements of vertical administrative contracting and horizontal promotion tournaments in the Chinese government system shape the lack of effective incentives for local officials to govern the environment [5,6]. Even after the central government strengthened environmental accountability and after the “one-vote veto” of environmental governance came to directly determine the probability of the promotion of local officials, environmental improvement remained lower than expected, and the strict performance assessment indicators did not provide effective incentives for the environmental governance of local governments. Rather, they caused distorted incentives for local officials to manipulate statistics. In the eyes of the local officials, indicators are just a numbers game [7,8]. Due to the lack of effective organizational incentives, the local environmental departments strictly enforce the law, thus allowing businesses to influence the environmental regulatory behavior of local governments through political connections such as lobbying and contributing tax and employment opportunities.

The policy effect of environmental regulation stems from the punitive effect of environmental enforcement on polluting behavior. Deterrence theory suggests that certainty and severity are the two dimensions that are important for achieving the deterrent effect of punishment, with certainty referring to the probability of being punished for the same violation, and severity referring to the degree of punishment for the same violation [9,10]. Certainty is a prerequisite for severity, and further discussion of the level of punishment becomes possible only if the perpetrator of the crime is caught for his illegal actions. In practice, increasing both the certainty and the severity of environmental regulations can achieve policy effects that reduce environmental pollution. On the one hand, an increase in the number of enforcement officers can increase the probability that violations are punished, and the number of environmental regulators is often negatively related to the level of emissions of regional enterprises. Alternatively, the adoption of advanced monitoring equipment or the implementation of new programs can improve the efficiency of environmental enforcers in performing their duties and can also achieve the policy effect of reducing environmental pollution. On the other hand, imposing taxes and fines on environmental pollution can also improve environmental quality. For example, in the fight against air pollution, strict environmental regulatory policies and political incentives have contributed to the achievement of the policy goal of a 10% reduction in sulfur dioxide during the period of the Eleventh Five-Year Plan [11,12]. The adoption of extraordinarily stringent environmental regulations is the main way of dealing with the water pollution crisis, and such strict regulations can mobilize concentrated resources and attention to achieve control results in the short term [13]. Government regulation is an effective tool that can be used to address the negative externalities of environmental pollution [14], and the ability and strictness of local government environmental regulation is a key factor in determining the environmental behavior of enterprises [15].

Similarly, regulatory space theory assumes that an effective regulatory system needs regulatory power and resources. However, the effectiveness of regulation can be de-creased by a desperate need for resources, and the regulated enterprises can also affect environmental regulation through their information advantage and social networks. In terms of micro-mechanisms, once the regulated enterprises have gained political connections by virtue of the informal power and resources they possess, regulators usually help companies to evade pollution penalties by issuing false regulatory reports [16]. Delayed deadlines and lower standards are becoming common phenomena, which inevitably have a weakening effect on the deterrent effect of environmental regulation.

According to deterrence theory, both the certainty and severity of punishment can achieve regulatory effects; thus, how do the policy effects of certainty and severity change once firms have political connections? Existing research does not provide a clear answer to this question. This paper tests this theoretical proposition using data from the 12th China Private Enterprise Survey in 2016. The main innovations of this paper are as follows. First, although existing studies have focused on the deterrent effect of punishment, they tend to replace the deterrent effect with severity, neglecting the focus on certainty, which is delineated in this paper, and considering severity as a mediating variable of the certainty effect. Secondly, this paper identifies the mechanisms through which political connections weaken the effects of regulation. The political connections of firms do not weaken the policy effects of regulatory certainty but can significantly weaken the policy effects of regulatory severity. Thirdly, existing studies tend to measure policy effects by objective indicators, which cannot avoid the interference generated by other factors, and their conclusions may be inaccurate for this reason. In fact, environmental regulation can lead to “greenwash” behavior by firms [17] seeking legitimacy through symbolic rather than actual substantive environmental practices [18]. In this paper, the policy effect of environmental regulation is based on the financial investment of enterprises in pollution control, which is the most direct manifestation of their environmental improvement behavior. Therefore, the conclusions of this paper are more realistic.

**Figure 1:**
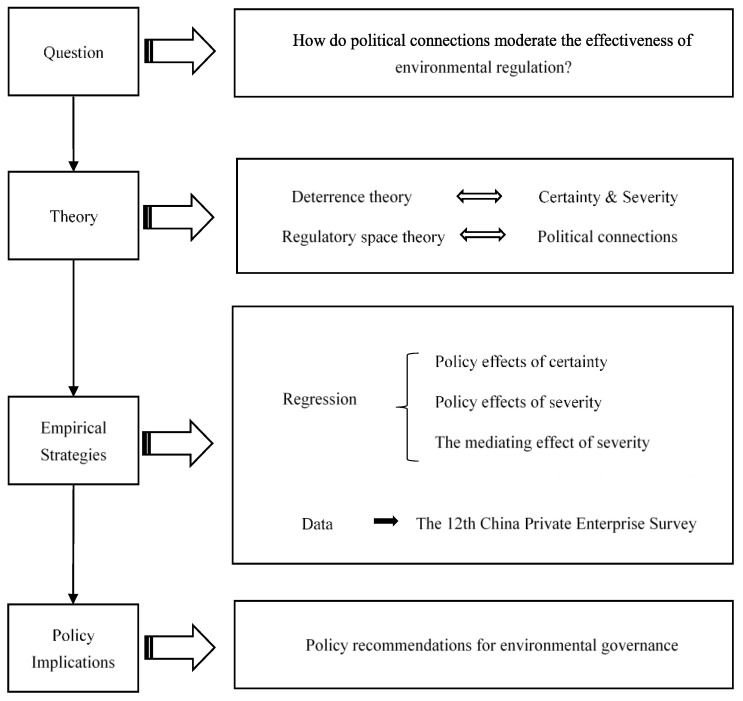
Research design.

The rest of this paper is structured as follows: the second part presents the research framework and research hypotheses based on deterrence theory and regulatory space theory; the third part presents the research design; the fourth part presents the results of the empirical analysis, which mainly tested the mediating effect of severity and the moderating role of political relations; and the fifth part concludes with a discussion. For a clearer understanding of the flow of the article, we provide a research design figure, which is shown in Figure 1.

## 2. Theory and Hypothesis

### 2.1. Deterrence Theory and Regulatory Behavior

The essence of regulation is that a prohibited behavior does not occur. However, compliance with the rules is never taken for granted. Why are individuals reluctant to follow the rules? Becker described choice behavior in a general sense from the perspective of utility maximization and illustrates this process through three propositions: (1) eco-nomic agents seek to maximize utility, which can be either an economic factor in the market or a non-monetary factor, such as dignity or prestige; (2) the basic preference types of individuals are relatively stable and do not undergo large dynamic fluctuations in the short term; and (3) the utility maximization motive is based on market choice, where the market adjusts the behavior of the participants to facilitate the realization of preferences through the allocation of resources [9,19]. In short, utility maximization, preference stability, and market equilibrium combine to shape individual behavioral choices. After specifying this idea from generalized choice behavior to regulatory or legal compliance behavior, Becker found that, when the expected benefits gained from performing a behavior prohibited by a rule exceed the benefits gained from devoting time and resources to engaging in other behaviors, rational individuals will perform that behavior regardless of whether they have the same risk preferences. Therefore, efficient public policy should raise the actor’s cost of performing a rule. Efficient public policy should therefore raise the cost to the actor performing a behavior prohibited by a rule in order to reduce the benefit gained by performing that behavior [10,20].

How, then, can one improve the effectiveness of regulation? The effectiveness of regulation depends primarily on two behavioral relationships: the cost of punishment and the elasticity of the offender’s response to arrest and conviction. For the same violation, differences in efficiency often stem from the elasticity of the response. Conversely, differences in efficiency are determined by the cost of arrest and conviction for different types of violation [10]. In terms of strategy choice, three strategies can improve the effectiveness of regulatory policies. The first is to reduce the cost of arrest and conviction: applying fingerprint technology, chemical detection, and other types of technology can reduce the cost of apprehension and increase the likelihood that the perpetrator will be punished. The second strategy is to increase the elasticity of the offender’s response to punishment: punishment methods such as imprisonment have a greater deterrent effect than monetary fines. The third strategy is timeliness: the time between the occurrence of an offense and its detection is also a factor that affects the effectiveness of regulation. The earlier the behavior is detected, the higher the likelihood is that the offense will be punished. In theory, all three strategies can reduce the benefits obtained from violations of regulatory requirements. In practice, increasing the likelihood and severity of the behavior being punished is the primary strategy. Thus, the deterrent effect of punishment reduces the expected benefits of the individuals committing such acts, and the effectiveness of regulatory policies depends on the certainty and severity of the regulation, the former being the likelihood of arrest and conviction, and the latter being the severity of the punishment following conviction.

On the one hand, improving regulatory certainty can achieve good regulatory effects. In general, there are two ways to increase the probability that a violation of a regulatory requirement will be punished. One is to increase the number of law enforcement officers. An increase in the number of police officers in an area can reduce the crime rate [21], and the number of environmental enforcers can also reduce the amount of emissions from businesses [15]. The other way is to change the scope of deployment and the means through which enforcers perform their duties without increasing the number of enforcers, which can also significantly improve the effectiveness of regulation [22]. For example, more advanced monitoring equipment and technology can help to obtain more effective information about an enterprise’s emissions; thus, the distance to the monitoring station in the field becomes a key factor influencing an enterprise’s emissions behavior [15,23]. Of course, the policy effect of improving the environment cannot be achieved if there is no deterministic deterrent effect on the discharge behavior of enterprises. In China, national monitoring stations are often set up in heavily polluted industrial areas. Large- and medium-sized enterprises that are strictly regulated do not pollute secretly at night because they face strict environmental monitoring, while small enterprises are more likely to engage in frenzied clandestine emissions under the cover of night [23]. In addition, the fundamental reason for the failure of the environmental subsidy program implemented by the Chinese government to motivate companies to participate in environmental management is the inadequacy of the existing environmental enforcement force [17]. Accordingly, this paper proposes Hypothesis 1:

**H1:**  *Certainty enhances the policy effectiveness of environmental regulation.*

On the other hand, increasing the severity of regulation is also an effective way to achieve the effect of environmental regulation. Many development practices show that strict environmental law enforcement is key to the improvement of environmental quality. Strict environmental law enforcement can act as a significant deterrent of the discharge behavior of enterprises, and after experiencing strict environmental penalties, both the illegal discharge behavior of enterprises and the number of illegal discharge enterprises show a decreasing trend [24,25]. For example, in the fight against air pollution, strict environmental regulatory policies and political incentives have contributed to the achievement of the policy goal of a 10% reduction in sulfur dioxide during the period of the Eleventh Five-year Plan [11]. Adopting extraordinarily stringent environmental regulatory measures is the main way of dealing with the water pollution crisis, and such strict regulatory measures can mobilize concentrated resources and attention in the short term to achieve control results [13]. What is more, strict environmental enforcement produces transitional compliance with the rules. Studies have shown that, when environmental agencies impose fines, companies with emissions that are already below the legally allowed standards reduce them further. In contrast, companies that are not in compliance cut their emissions to meet the penalties well beyond the legal standards; thus, the policy effects of severity are far greater than expected [26]. Accordingly, this paper proposes Hypothesis 2:

**H2:**  *Severity can improve the policy effectiveness of environmental regulation.*

Theoretically, certainty and severity form the policy toolset for inducing compliance with respect to corporate behavior, and environmental regulation thus includes basic administrative inspections and penalties for polluting behavior. Regarding the relationship between the parties, certainty is a prerequisite for severity. Further discussion of the degree of punishment becomes possible only if the perpetrator of the crime is caught for his illegal act [27]. Thus, the effectiveness of environmental regulations depends on the extent to which they are enforced, and the decoupling of environmental regulation from enforcement can weaken the influence of regulation on corporate environmental behavior [28]. For example, the new ambient air quality standards, originally introduced to improve air quality, did not have the desired effect in provinces where enforcement was weak [29]. Moreover, rather than being a hierarchical system of rules that are uniformly implemented and enforced, environmental enforcement is a coordination mechanism that interacts regularly with market pressures, local governments, environmentalists, and corporate management culture [30], with different interactive processes capable of producing different policy effects. In this sense, therefore, the severity of the penalty determines the policy effect of the entire environmental regulation process. In general, certainty can have a deterrent effect provided that the regulatory system can effectively identify violations and impose penalties: the higher the degree of severity, the more credible the promise of certainty. If a company’s violations are not strictly punished, the probability that the violations will be punished is insignificant. Therefore, under a complete environmental regulation system, severity is a mediating variable of certainty. The higher the degree of environmental regulation certainty is, the higher the severity of the punishment and the stronger the binding force on enterprises’ emission behavior will be. Therefore, we propose Hypothesis 3:

**H3:**  *Firms with higher deterministic pressure to regulate are subject to higher severity punishment.*

### 2.2. Regulatory Space Theory and Regulatory Effects

The deterrent effect of punishment relies on the accountability of the regulatory process, but the fragmented distribution of resources can also result in regulatory capture, which, in turn, weakens the accountability of environmental regulation. Regulatory space theory suggests that effective regulation requires the possession of financial, human, information, and other organizational resources, but that these resources are fragmented and distributed between the regulator and the regulated. The regulator is unable to achieve monopoly possession of resources, as the regulated enterprises have a greater advantage in terms of information resources and can therefore gain greater bargaining power. This informal power has a significant impact on the entire regulatory process [31]. Logically, when a firm’s violation is identified by a regulator, the firm can either accept the penalty by paying a fine, or it can evade environmental regulation by paying a fee to collude with the regulator. In China, environmental enforcement is neither adequate nor effective in the local sector. On the one hand, due to factors such as environmental values, the perceived organizational capacity for enforcement, and the level of government support for environmental protection, local officials have a biased understanding of the effectiveness of enforcement, resulting in little success in environmental enforcement by local environmental departments [32]. On the other hand, the network of relationships emphasized in Chinese cultural values increases the flexibility of environmental enforcement, and the bargaining power of firms affects the performance of environmental enforcement authorities in collecting sewage charges, with state-owned firms having more political connections and higher bargaining power than private firms [33]. Thus, in a multilayered bureaucracy, firms have political connections that can regulate the enormous impact of their behavior. The ability of firms to influence the regulatory behavior of local governments through contacts, bribes, or favors and the ability of localities to use their discretionary power to protect these politically connected firms [34], which results in bureaucratic or regulatory capture, suggest that environmental regulation by local governments does not rely on their own formal authority, but rather on the decisions made by the firms that are supposed to be regulated [35,36]. Thus, the effectiveness of environmental regulations depends on the specific ways in which they are implemented in practice, and the political connections that companies have are a key factor in the enforcement of regulations. In terms of regulatory strategy, the effectiveness of central environmental inspectors lies in their ability to break the influence of corporate political connections on local environmental regulation, which, in turn, can strengthen the effectiveness of local environmental regulation [37,38].

In fact, in the transition period in China, companies are often keen to reduce the impacts of mandatory regulations by acquiring political connections, often through tax and employment contributions, so as to gain the popularity or approval of local officials and, thus, the opportunity to become deputies to the National People’s Congress or members of the Chinese People’s Political Consultative Conference at various levels. Once such political connections have been obtained, companies are able to use them (the political connections do not represent a status) to influence the policy-making process of local governments or to avoid penalties in the policy implementation process. Political connections can moderate the deterrent effect in two ways. On the one hand, political connections can moderate the deterministic policy effect, as firms with more political connections are less likely to be subject to environmental fines and will be fined less. On the other, political connections can also influence the effect of harsh policies, as firms with political connections are more reluctant to change their emission behavior, even when faced with the same amount of fines as other firms without political connections. Thus, firms with political connections can influence the effects of regulation, and this influence can be achieved by moderating the effects of deterministic and severe policies. Accordingly, we propose Hypotheses 4 and 5:

**H4:**  *Political relations have a weakening effect on the punitive effect of regulatory certainty.*

**H5:**  *Political relations have a weakening effect on the pollution control effect of regulatory severity.*

## 3. Methods and Data

### 3.1. Data Sources

The research sample in this paper is individual private enterprises, and the data come from the China Private Enterprise Survey conducted by the Central United Front Work Department, the All-China Federation of Industry and Commerce, the State Administration of Market Supervision, the Chinese Academy of Social Sciences, and the Private Enterprise Research Group of the China Private Economy Research Association (CPES). The survey is conducted every two years, and the data used in this paper are from the survey conducted in 2016, which were obtained upon application through the official website. The survey used the national list of private enterprises provided by the State Administration for Industry and Commerce as the sampling frame. A more standardized random sampling method was conducted, and the sample was deemed highly representative and applicable to the research questions in this paper. According to the variables related to this paper, a total of 8111 valid samples were obtained.

### 3.2. Variables

#### 3.2.1. Dependent Variable

The dependent variable is the effect of environmental regulation. Existing measurements of the effects of environmental regulation have been conducted in two main ways. The first is the use of the environmental quality index of each region as a measure, which effectively ignores the fact that changes in environmental quality are the combined result of many complex factors. The second way is to measure the environmental behavior of enterprises but to select objective criteria, such as the enterprise’s environmental self-assessment report, in regard to the question of whether environmental certification has been obtained. This measurement does not actually reflect the environmental pollution behavior of enterprises, and this environmental behavior, as a symbolic response of the enterprises, does not really affect the dis-charge behavior of the enterprises [17,39]. Therefore, in terms of operationalization, we selected the direct environmental behavior of enterprises as the measurement method (that is, the environmental pollution capital investment of enterprises). Question 40 of the questionnaire asks about the financial investments made by companies in fighting environmental pollution in 2015, for which we assigned the following categories: if the enterprise invested in environmental pollution control in 2015, the value is assigned as 1; otherwise, the value is assigned as 0. The main reason for selecting the operationalization of the fixed class variables is that it is more important to consider whether the enterprise shows the behavior of managing environmental pollution than to consider the difference in the degree of environmental pollution managed by the enterprise.

#### 3.2.2. Independent Variable

The independent variable is the certainty of environmental regulation. Existing studies tend to measure the certainty of regulated behavior in terms of the number of enforcers in each region [10,27], a measure that implies that different firms in the same region face the same degree of regulatory certainty, but this is clearly not the case. For this reason, we decided to measure the certainty faced by firms by asking them directly in the questionnaire about their perceived environmental regulatory pressures. Question 42 of the questionnaire asks, “How much pressure does government environmental regulation put on your business to protect the environment?”, with options ranging from “very much pressure”, “a lot of pressure”, “average pressure”, and “little pressure” to “no pressure”, each assigned a score of 5–1.

#### 3.2.3. Mediating Variable

Regulatory severity is the mediating variable in this paper. Existing studies usually measure the severity of punishment. For example, the death penalty rate also measures the severity of criminal behavior. Drawing on existing studies, we measure the regulatory stringency faced by firms in terms of the number of fines they receive for environmental pollution. The second question in Questionnaire 40 is used to determine this: “How much did your company spend on environmental pollution in 2015?” To ensure sample smoothness, we normalized the results by using the natural logarithm after adding 1 to all of the values.

#### 3.2.4. Moderating Variable

The moderating variable is the political affiliations of private firms. There are two main ways of measuring political relations: one is to measure the amount of money a company spends on public relations [40], which may be underreported by the company to conceal the actual political relations, making the actual value greater than the value reported by the company; and the other is to measure the political status of enterprise managers, which is measured based on the question of whether the enterprise managers hold public office. We selected this method of operationalization. Specifically, we measured this using Question 8 of the questionnaire: “Are you a member of the National People’s Congress or the Chinese People’s Political Consultative Conference?” We used this variable as a dummy variable, with companies with managers who have served as NPC deputies or CPPCC members being assigned a value of 1. Otherwise, they were assigned a value of 0.

#### 3.2.5. Controlling Variables

In addition, we added the necessary control variables, including the nature of the enterprise (whether it is listed), the efficiency of the enterprise (the ratio of net profit to operating income), the nature of the industry (whether the enterprise’s first main business is in mining, manufacturing, and construction, or water, electricity, and gas supply), the capital composition (whether there is foreign capital or capital investment from Hong Kong, Macao, and Taiwan), the financial environment (how difficult it is for the enterprise to raise funds from the private sector), and the status of the industry (whether local government leaders visit the enterprise or work on-site). The descriptive statistics and results of the specific variables are shown in Table 1.

### 3.3. Methods

This paper examines the mediating effect of regulatory severity and the moderating role of political relations, and the empirical model constructed is as follows:

(1) $$m=a_{0}+a_{1}x+a_{2}w+a_{3}xw+\varepsilon$$

(2) $$y=b_{0}+b_{1}m+b_{2}x+b_{3}w+b_{4}xw+b_{5}mw+\varepsilon$$

where *y* denotes the firm’s capital investment in environmental pollution control, *x* is the degree of certainty of environmental regulation, *m* is the degree of severity of environmental regulation, and *w* indicates whether the firm has political connections. Equation (1) focuses on the effects of regulatory certainty and political relations on regulatory severity. Equation (2) is concerned with the moderating effect of political relations on the mediating effect of regulatory severity.

## 4. Results and Discussions

### 4.1. Environmental Regulation and Corporate Pollution Cost Inputs: The Mediating Role of Severity

We used multiple regressions to test the policy effects of environmental regulation empirically. First, we tested the mediating role of severity, and the results are shown in Table 2: the certainty pressure on firms can increase the severity of punishment for the environmental pollution behavior of firms, while at the same time, severity can encourage firms to increase their corporate pollution control investment and, of course, the same policy effect can be obtained with certainty. The certainty and severity of environmental regulations explain 19.9% of the behavior of the firms in managing environmental pollution without considering the effects of the control variables, and this share increases to 21.5% when considering the effects of the relevant control variables. This suggests that the severity of environmental regulations is indeed one of the mediating paths through which certainty achieves policy effects, i.e., the higher the certainty of environmental regulations is, the higher the severity of the penalties faced by firms is, and the more willing they are to undertake efforts to combat environmental pollution.

In addition, we performed the bootstrap test and obtained the following results after repeating the sampling 500 times. From the test results shown in Table 3, the total effect of environmental regulation on the financial investment behavior of enterprises in combating pollution is 0.08; the direct utility is 0.075; the mediating effect of the severity of environmental regulation accounts for 6.685% of the total effect; and the 95% confidence interval does not reach 0. This indicates that the mediating effect of severity is significant and robust, and the hypothesis of the mediating effect is verified.

### 4.2. Environmental Regulation and Corporate Pollution Cost Inputs: A Mediating Role in Regulation

We focus on the moderating role of political relations in influencing the mediating effects of environmental regulatory certainty. Models 1 and 2 are the main models, and Models 3 and 4 are the results after adding the control variables. The study’s results (Table 4) found that regulatory certainty can significantly increase the severity of penalties and, at the same time, promote the financial investment of enterprises in order to combat environmental pollution. H1 and H3 are valid. The severity of the regulation can also achieve the policy effect of increasing the investment of enterprises in pollution control. H2 is valid. Regarding the moderating role of political relations, political relations contribute to the punitive effect of regulatory certainty, and the two are positively causally related (β = 0.029, *p* < 0.01). Compared to firms without political connections, firms with political connections have a higher degree of perceived certainty of environmental regulation and a higher degree of the severity of environmental regulation penalties. In contrast, political connections have a weakening effect on the pollution control effect of the severity of environmental regulations, and the two are negatively causally related (β = −0.107, *p* < 0.01), which indicates that firms with political connections invest less in environmental pollution control in the face of environmental penalties relative to firms lacking political connections. Therefore, H4 is not valid, and H5 is valid.

As for the control variables, the listed companies are more willing to invest costs in environmental pollution treatment. Corporate private financing is easier and, in this way, enterprises more willing to carry out environmental pollution treatment. Compared with other industries, enterprises that are mainly industrial are more likely to invest in pollution control. Likewise, enterprises at the forefront of the industry have the will to control environmental pollution. On the contrary, the capital composition and economic effects of the firm do not affect the firm’s capital investment in environmental pollution. These findings are consistent with the conclusions of existing studies [40,41].

**Figure 2:**
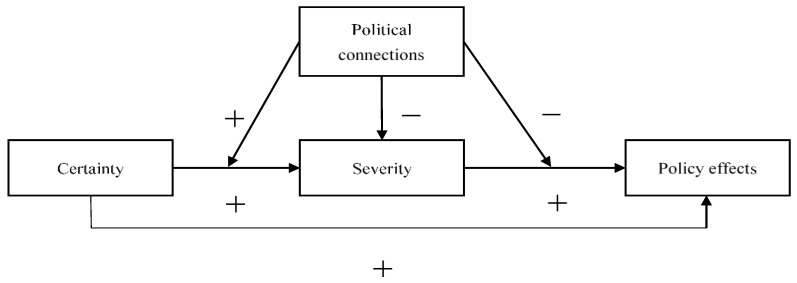
Schematic diagram of the mediated results with respect to regulation.

From a cybernetic perspective, the deterrence effect depends on the effectiveness of the regulatory system, while an effective regulatory system consists of three parts: target values, monitoring and feedback mechanisms, and adjustment mechanisms. Of these, the target value reflects some standards and norms through which the system needs to operate, and the formulation of rules is the direct embodiment of the target value. Monitoring feedback is expressed as supervision and inspection during the system’s operation, emphasizing the effective identification of violations, which is the core of the entire regulatory system. Adjustment mechanisms, on the other hand, are the sanctions imposed when the system deviates from its goals and, generally, when behaviors that are judged to violate regulatory requirements receive penalties [42]. Throughout the regulatory process, the gain obtained by the actor by violating the regulatory requirement is determined by the deterrent effect of the regulatory policy, and the actor, who risks performing the act, is a combined function of the perceived probability of being punished and the perception about the magnitude of the punishment, an effect that is simultaneously moderated by the political relations possessed by the actor. To show more clearly the causal relationship between the variables, we plot the results of the mediating role of severity and the moderating role of political relations in Figure 2. Specifically, the three causal relationships identified in this paper are as follows. (1) Both certainty and severity can achieve the policy effects of environmental regulation, which is consistent with the basic proposition of deterrence theory [10,27]. (2) The political relations that firms have can significantly moderate the policy effects of environmental regulation and can significantly reduce the severity of the penalties for corporate violations, which accords with regulatory space theory [31,34]. (3) Political relations exert different moderating effects on certainty and severity, respectively, enhancing the policy effects of certainty and weakening those of severity, a finding which supports the deterrence and regulatory capture theories.

The previous empirical results show that political relations moderate the mediating effect of environmental regulatory severity. This suggests that the mediating effect of regulatory severity varies across degrees of political relations. To demonstrate the moderating effects of political relations on the mediating variables more clearly, we examined the mediating effects of the moderating variables under three different conditions: the mean, mean − 1 standard deviation, and mean + 1 standard deviation. The results are reported in Table 5. The results show that the mediating effect of environmental regulation severity has a positive relationship with the value of the political relationship; that is, the conditional indirect effect increases with the increase in the moderating variable.

Finally, we used the bootstrap method for robustness testing, and the following results were obtained after 500 repetitions of sampling. Table 6 shows that the mediating effect of regulatory severity and the moderating effect of political connections on the mediating effect are both significant, and the 95% confidence interval does not include a value of 0. This indicates that the causal relationships between the variables found in this paper are robust.

## 5. Conclusions and Recommendations

### 5.1. Conclusions

In this paper, we used data from the 12th Chinese Private Enterprise Survey in 2016 to answer the theoretical question of how political relations affect the policy effects of environmental regulation. The main finding of this paper is that severity is a mediating variable of environmental regulations aiming to promote the investment of firms in pollution control, but this mediating mechanism is moderated by the political relations of firm managers. In theory, political relations can influence the effects of regulation on the two dimensions of certainty and severity, but this is not the case in reality. First of all, as described in deterrence theory, both certainty and severity can achieve the policy effect of environmental regulation. This conclusion deepens the explanatory range of deterrence theory. Secondly, just as regulation space theory emphasizes, the informal political resources owned by enterprises can also significantly affect the regulation effect, and this influence process is mainly realized by reducing the severity of the punishment. To make clear the fact that this is the third item in a numbered list, in this paper, we further identified the moderating effect of political relations on the mediation mechanism. Consistent with our theoretical expectations, political relations weaken the policy effect of regulatory severity on firms’ efforts to combat environmental pollution. Although fines for environmental pollution can increase costs, firms lack the incentive to combat pollution if they can still gain from their emissions, and political relations further weaken the incentive. Contrary to our theoretical expectations, political connections instead increase the policy effect of regulatory certainty, implying that increasing the degree of regulatory certainty is more effective for firms with more political connections than for those without. Thus, the influence of political relations on the policy effects of environmental regulation is realized through the behavioral bias of enterprises working to “avoid real harm but do false good”. In terms of external behavior, firms with political connections are not significantly less likely to be subject to environmental fines, but environmental fines have a diminished role in promoting improved environmental quality. Enterprises prefer to pay environmental fines, which is a “false good” environmental management behavior, and try to avoid increasing capital investment, which is a “real harm” environmental management behavior.

Why do political relations moderate the effects of deterministic punishment and the effects of harshness differently in the treatment of pollution? We argue that the answer to this question is determined by the question of whether the behavior has an external character. In China, administrative penalties are subject to public disclosure, which means that the severity is clearly visible, but the policy effects of severity (the behavior of firms in reducing pollution) are not clearly visible. In theory, companies with political connections should be subject to less severe environmental fines, but our study found otherwise. The negative externality brought by the environmental pollution of enterprises triggers the environmental participation of residents in nearby communities. If enterprises are fined less because of their political connections, this is equivalent to publicly declaring the failure of environmental regulation because of political connections, which is a result that no local environmental law enforcement department can bear. Therefore, for enterprises with political connections, the law enforcement departments increase punishment after obtaining clear evidence of violations so as to respond to the environmental participation of the masses. Administrative punishment has obvious externalities. On the contrary, the decision regarding whether companies use this as an opportunity to invest in pollution control after they have been punished is an internal behavior of the companies, and the community and the public do not have information about the daily production and operation of the companies once they move. Therefore, those companies with political connections lack a strong incentive to actively invest costs in order to control environmental pollution. It follows that, for firms with political connections, local environmental enforcers are motivated to address the negative externalities of environmental problems through administrative penalties rather than to restrain the environmental pollution behavior of firms. The former has visibility and can respond more effectively to the public’s demand for the negative externalities of environmental pollution, while the latter does not have this function, thus prompting companies to adopt an “avoid real harm but do false good” strategy.

In a multiple-structure environmental regulatory system, it is often difficult for grassroots environmental enforcement departments to strictly enforce the law [30], and the policy burdens of taxation and employment borne by enterprises often mean that local governments choose to allow or even accommodate the environmental pollution behaviors of enterprises, thus creating a “law enforcement deviation” effect, in which the policy implementation deviates from the policy objectives, and the implementation results are inconsistent with the policy issues. In other words, the enforcers use their discretionary power to cause the original regulatory behavior to deviate from the initial mandate and gain other benefits by reinterpreting the policy objectives [43]. Deterrence theory treats certainty and severity as two equal policy tools [10,27], but certainty is only effective through severity, and only strict punishment for polluting behavior can ensure that the certainty of punishment is credible.

### 5.2. Policy Recommendations

The main policy recommendations of this paper are as follows. (1) As deterrence theory emphasizes that the certainty and severity of punishment can reduce the benefits of violations [10,27], without considering the influences of corporate political relations and policy costs, improving the certainty and severity of environmental regulations can achieve effective regulation. Specific measures can be taken in the following two ways. On the one hand, the ability of enforcers to identify corporate emissions can be improved by increasing the number of environmental enforcers or by adopting advanced equipment. On the other, the severity of environmental regulations can be achieved by increasing the penalties for corporate emissions. (2) Given the influence of corporate politics, reducing the influence of corporate political relations on the effectiveness of environmental regulation is also in line with the basic proposition of regulatory capture theory [31,34]. Environmental regulation can be carried out by introducing independent third parties, which can break the original government–enterprise relationship and reduce the interference of corporate political relations in the effectiveness of environmental regulation. The central environmental protection inspectors employed by the Chinese government for this purpose are an effective way of achieving this goal, and their work should be continued. On the other hand, the enforcement of environmental regulation in different places can be implemented, which can also reduce the influence of corporate political relations on the effect of environmental regulation. (3) Of course, the two approaches mentioned above require additional enforcement resources for environmental regulation, and given the limited law enforcement resources and the cost of policy implementation, it is often difficult for grassroots environmental departments to achieve both certainty and rigor. How, then, can one maximize the efficiency of environmental regulation? The authors of this paper believe that improving the severity of environmental regulation is the best strategy. The firm’s political connections do not reduce the penalties imposed on the firm. Thus, certainty is effective, but political connections can reduce the increased capital investment of companies due to environmental penalties. Therefore, improving the effectiveness of the severity of punishment can obtain better policy effects than certainty.

## Figures and Tables

**Table 1:** Operationalization of the variables and descriptive statistics.

Variable	Operationalization	Observations	Mean	Std	Min.	Max.
Invest	Is there a cost input for environmental pollution control? (1 = yes)	8111	0.342	0.474	0	1
Severity	Has paid the number of environmental pollution fines (log)	7030	0.04	0.331	0	8.007
Certainty	Environmental pressure on the business from government environmental regulations	7610	2.494	1.294	1	5
Politics	NPC deputy or CPPCC member? (1 = yes)	8111	0.239	0.426	0	1
Listed	Is it a listed company? (1 = yes)	8111	0.02	0.14	0	1
Industry	Is the enterprise’s first main business mining, manufacturing, and construction, or utilities? (1 = yes)	8111	0.375	0.484	0	1
Benefit	Enterprise net profit to operating income ratio	6590	0.122	0.331	−1.5	1.5
Money	The degree of difficulty for companies to raise funds from private sources	7497	2.587	0.969	1	5
Foreign	Does the enterprise have foreign capital or capital investment from Hong Kong, Macao, or Taiwan? (1 = yes)	8111	0.399	0.49	0	1
Inspect	Have local government leaders visited the company? (1 = yes)	8111	0.493	0.5	0	1

**Table 2:** Results of the analysis of the mediating role of severity.

	Model 1	Model 2	Model 3
	Invest	Severity	Invest
Certainty	0.081 ***	0.027 ***	0.076 ***
	(0.004)	(0.003)	(0.004)
Listed	0.129 ***	−0.022	0.133 ***
	(0.039)	(0.03)	(0.038)
Industry	0.203 ***	0.019 **	0.199 ***
	(0.012)	(0.009)	(0.011)
Benefit	−0.037 **	−0.014	−0.034 **
	(0.016)	(0.012)	(0.016)
Money	0.026 ***	0.005	0.025 ***
	(0.006)	(0.004)	(0.006)
Foreign	0.010	0.011	0.008
	(0.012)	(0.009)	(0.011)
Inspect	0.181 ***	0.025 **	0.176 ***
	(0.011)	(0.009)	(0.011)
Severity			0.186 ***
			(0.017)
_cons	−0.163 ***	−0.062 ***	−0.151 ***
	(0.02)	(0.015)	(0.02)
Observations	6771	6771	6771
F	215.91 ***	9.778 ***	233.683 ***
R-squared	0.199	0.019	0.215

Standard errors in parentheses, *** *p* < 0.01, ** *p* < 0.05. It is same below.

**Table 3:** Bootstrap test results of the mediation effect.

	Observed	Bootstrap	z	Normal-Based	
	coef.	Std Err.		[95% Conf. Interval]	
_bs_1	0.005 ***	0.001	6.27	0.003	0.007
_bs_2	0.076 ***	0.005	16.76	0.067	0.085

*** *p* < 0.01.

**Table 4:** Mediated outcome tests with respect to moderation.

	Model 1	Model 2	Model 3	Model 4
	Severity	Invest	Severity	Invest
Certainty	0.018 ***	0.068 ***	0.021 ***	0.063 ***
	(0.003)	(0.004)	(0.004)	(0.005)
Politics	−0.048 **	0.125 ***	−0.058 ***	0.074 ***
	(0.019)	(0.026)	(0.022)	(0.028)
Politics × Certainty	0.029 ***	0.049 ***	0.024 ***	0.039 ***
	(0.007)	(0.009)	(0.007)	(0.010)
Severity		0.247 ***		0.203 ***
		(0.021)		(0.021)
Politics × Severity		−0.107 ***		−0.066 *
		(0.034)		(0.037)
Listed			−0.023	0.105 ***
			(0.030)	(0.037)
Industry			0.018 **	0.188 ***
			(0.009)	(0.011)
Benefit			−0.014	−0.021
			(0.012)	(0.016)
Money			0.005	0.016 ***
			(0.004)	(0.006)
Foreign			0.012	0.002
			(0.009)	(0.011)
Inspect			0.025 ***	0.141 ***
			(0.009)	(0.011)
Observations	6771		5421	
R^2^	0.09	0.164	0.098	0.157

*** *p* < 0.01, ** *p* < 0.05, * *p* < 0.1.

**Table 5:** Results of the robustness tests of the moderating effect.

	Coef.	Std Err.	z	[95% Conf. Interval]	
Mean − 1 sd	0.003	0.001	3.16	0.001	0.006
Mean	0.006	0.001	7.39	0.004	0.007
mean + 1 sd	0.007	0.001	6.64	0.005	0.009

**Table 6:** Bootstrap test results for mediating effects with respect to moderation.

	Observed	Bootstrap	z	Normal-Based	
	coef.	Std Err.		(95% Conf. Interval)	
_bs_1	0.003 ***	0.001	2.90	0.001	0.006
_bs_2	0.006 ***	0.001	5.87	0.004	0.007
_bs_3	0.007 ***	0.001	5.99	0.004	0.009

*** *p* < 0.01.

## Data Availability

Not applicable.
